# Effect of Season, Transport Length, Deck Location, and Lairage Length on Pork Quality and Blood Cortisol Concentrations of Market Hogs

**DOI:** 10.3390/ani4040627

**Published:** 2014-09-29

**Authors:** David Newman, Jennifer Young, Chad Carr, Matt Ryan, Eric Berg

**Affiliations:** 1 Department of Animal Sciences, North Dakota State University, Fargo, ND 58108, USA; E-Mails: jennifer.m.young@ndsu.edu (J.Y.); eric.p.berg@ndsu.edu (E.B.); 2 Department of Animal Sciences, University of Florida, Gainseville, FL 32611, USA; E-Mail: chadcarr@ufl.edu; 3 Farmland Foods, Wichita, KS 67213, USA; E-Mail: mryan@farmland.com

**Keywords:** lairage, pork quality, season, swine, transport, welfare

## Abstract

**Simple Summary:**

Transport of hogs is a routine practice in the swine industry. Loading pigs onto the trailer, transporting them to the plant, and having them wait in an unfamiliar pen at the plant prior to slaughter are all stressful to the pigs. Seasonal changes in temperatures can also affect the amount of stress a hog is subjected to during transport to market. Therefore, the objective of this study was to investigate the effect of transportation and lairage conditions on stress, evaluated by measuring serum cortisol concentrations, and the effect on pork quality.

**Abstract:**

The objective of this study was to investigate the effects of seasonal environment, transport conditions, and time in lairage on pork quality and serum cortisol concentrations. Market hogs were slaughtered during winter (n = 535), spring (n = 645), summer (n = 644), and fall (n = 488). Within season, hogs were randomly assigned to treatments in a 2 × 2 × 2 factorial arrangement, with 2 deck locations (top *vs.* bottom) and 2 transport and lairage durations (3 h *vs.* 6 h). Blood samples were collected at exsanguination for analysis of cortisol concentration. Loins were collected at 24 h postmortem for pork quality assessment. Season and deck did not have a main effect on cortisol concentrations or pork quality. Hogs transported 6 h had increased cortisol concentrations (103.0 *vs*. 95.5 ng/mL; P < 0.001) and decreased L* (52.49 *vs.* 52.69; P = 0.09), b* (6.28 *vs.* 6.36; P = 0.03), and hue angle (20.70 *vs.* 20.95; P = 0.03) compared to hogs transported 3 h. Hogs subjected to 6 h of lairage had increased 24-h pH (5.69 *vs.* 5.66; P = 0.005), a* (16.64 *vs.* 16.48; P < 0.0001), b* (6.42 *vs.* 6.22; P < 0.0001), saturation (17.85 *vs.* 17.64; P < 0.0001), and hue angle (21.01 *vs.* 20.65; P = 0.002) and decreased L* (52.49 *vs.* 52.69; P = 0.07) when compared to hogs subjected to 3 h of lairage.

## 1. Introduction

In 1992, the estimated occurrence of pale, soft, and exudative (PSE) pork at the packing plant was 10.2% [1]; in 2002, it was reported to have increased to 15.5% and to have an economic loss of ~1% per carcass [2]. Selection for lean growth has been shown to increase the incidence of PSE pork [3]; therefore, it is important to understand other factors that increase the incidence of PSE pork. Loading, transport, and unloading have been identified as high stress events for hogs [4]. These pre-slaughter stressors increase metabolic functions, which may lead to reductions in many pork quality characteristics [5]. Today, there are two distinct trailer environments in the popular double-deck livestock trailers. Hogs in certain locations within these trailers are subjected to different microenvironments within the trailer. In broiler chickens, heterogeneous distributions of temperature and humidity within transport trailer have been shown to occur due to inadequate ventilation [6]. Although no differences were found between top and bottom decks, dalla Costa *et al.* [7] found that pigs transported in the rear of the trailer had more bruises at unloading than pigs transported in the front of the trailer. Pigs have been shown to become sick from traveling, whether the journey was smooth or rough and whether or not pigs were fasted before transport [8]. Therefore, the objective of this study was to determine the effects of seasonal environment, top or bottom trailer deck location, transport duration, and time in lairage on overall pork quality and serum cortisol concentrations of market hogs.

## 2. Experimental Section

### 2.1. Animals and Experimental Design

Commercial crossbred market hogs (n = 2,312; PIC, Franklin, KY, USA) were slaughtered on dates representing traditional seasonal environments in the Midwestern United States: February 14 and 16 (winter; n = 535), May 16 and 18 (spring; n = 645), August 1 and 3 (summer; n = 644), and October 17 and 19 (fall; n = 488) of 2006. Slaughter occurred on Tuesday and Thursday within the same week within each season. Within season, hogs were randomly assigned to 1 of 8 treatments in a 2 × 2 × 2 factorial arrangement, with 2 transport durations (3 h *vs.* 6 h), 2 trailer deck locations (top *vs.* bottom), and 2 lairage durations (3 h *vs.* 6 h). The authors worked with procurement at Hormel and a commercial producer to collect data.

**Figure 1:**
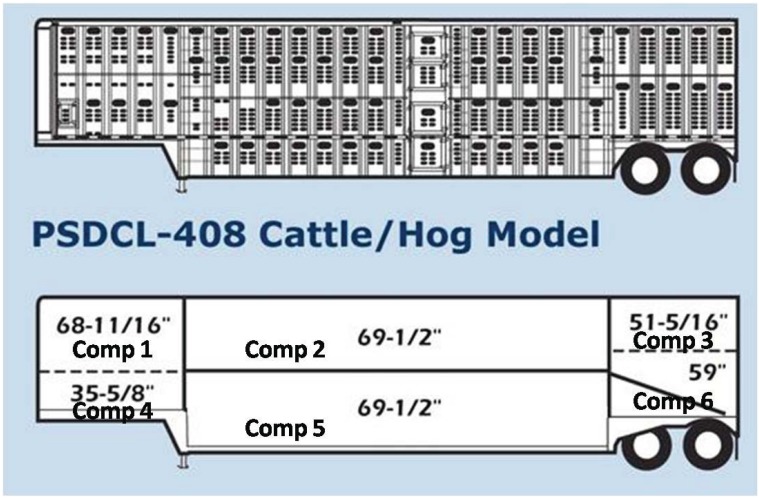
Experimental trailer design and compartment location within trailer (www.wilsontrailer.com).

**Figure 2:**
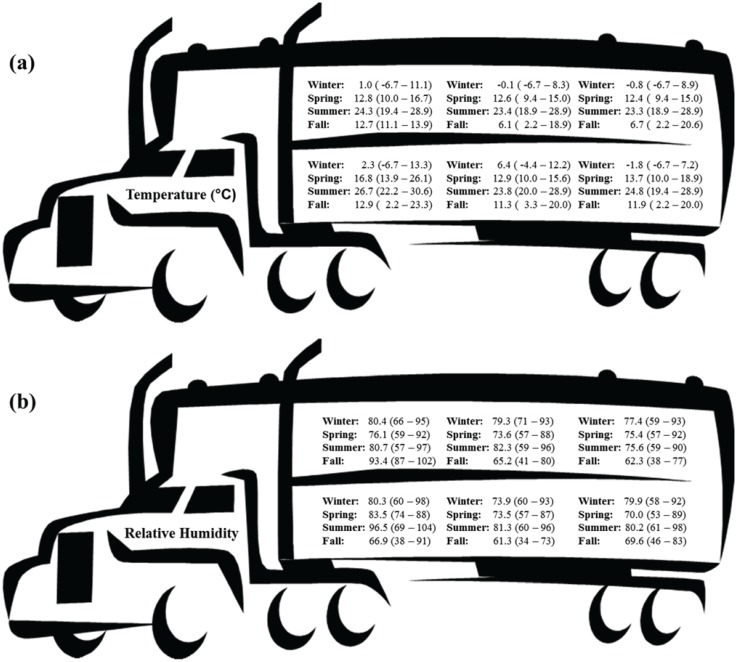
Averages and ranges of temperatures (**a**) and relative humidity (**b**) for each trailer compartment for each season of the 3 h transport duration.

**Figure 3:**
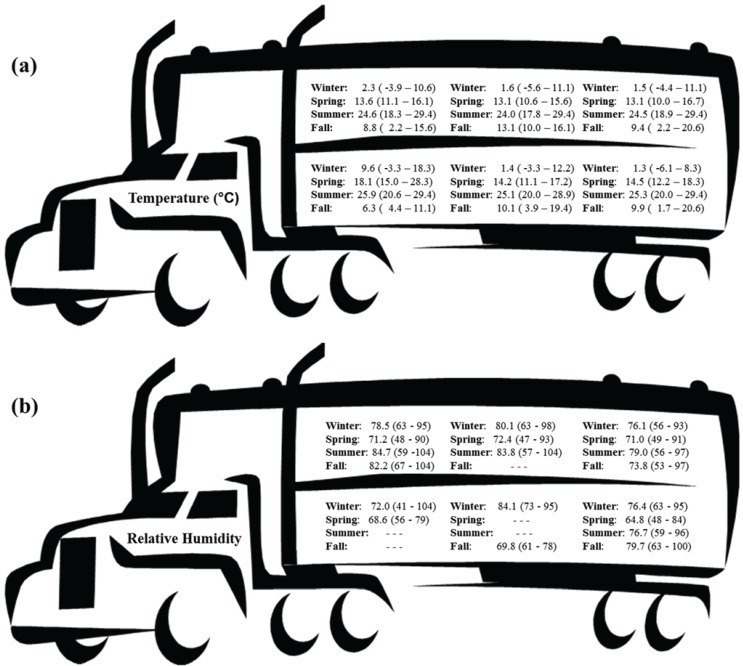
Averages and ranges of temperatures (**a**) and relative humidity (**b**) for each trailer compartment for each season of the 6 h transport duration.

The commercial producer was responsible for all loading procedures and was directed to load via standard operating procedures. All hogs were fed the same commercial diet and feed was withheld starting at 2100 h the night before slaughter. Hogs were loaded in groups of 20 with no use of electric prods, starting with the top deck first as typical of commercial loading. Hogs assigned to the 6 h treatment were loaded and left the farm at 2330 the night before slaughter while hogs assigned to the 3 h treatment were loaded and left the farm at 0200 the morning of slaughter (Table 1). Both truckloads of hogs slaughtered on the same day originated from the same barn. Trucks used for transport were pot-belly style (Figure 1). Environmental conditions (temperature and relative humidity) in the trailer were monitored at one minute intervals using portable data loggers (HOBO Pro Series RH/Temp. Onset Computer Corp., Pocasset, MA, USA) located in the three compartments (front, middle, rear) of both decks (Figure 2 and Figure 3). 

**Table 1:** Time schedule for pigs going to slaughterhouse.

▪Action	Time schedule
▪Loading pigs for 6 h transport:	2300–2330 h
▪Loading pigs for 3 h transport:	0130–0200 h
▪Arrival at slaughterhouse:	0500 h
▪Begin driving to stunner for 3 h lairage:	0755 h
▪Slaughter for 3 h lairage:	0800 h
▪Begin driving to stunner for 6 h lairage:	1055 h
▪Slaughter for 6 h lairage:	1100 h

All hogs arrived at the commercial packing plant (Quality Pork Processors, Austin, MN, USA) at 0500 h. Upon arrival, hogs transported for 6 h were unloaded first, beginning with the bottom deck. All hogs were unloaded in groups of 20 and alternately assigned to 3 h or 6 h lairage pens; therefore, pigs subjected to each of the 4 transport treatments (top and bottom decks for 3 h and 6 h transport durations) were comingled in lairage. Each hog in the small subgroup was tattooed with a number to specify transport duration, deck location, and lairage length. Hogs were given free access to water during lairage. All hogs were subjected to humane head-to-heart electrical stunning procedures in compliance with the standard industry-accepted practices and the Humane Slaughter Act [9]. Slaughter started at 0800 h for hogs in the 3 h lairage and at 1100 h for those in the 6 h lairage (Table 1).

At exsanguination, 15 mL of whole blood were collected from each hog in pre-identified disposable tubes (Nalge NUNC International, Rochester, NY, USA). Sequentially numbered, self-piercing, “round post” metal ear tags, corresponding with the blood collection tubes, were then inserted into the ear of each hog while on the gambrel table. After all blood samples were collected, blood tubes were allowed to clot under refrigeration (5 °C). Blood samples were centrifuged for 10 min at 2,500 × g between 8 and 10 h after collection. After centrifugation, 5 mL of serum were transferred into 48-well plates and stored at −20 °C until analysis for cortisol concentration. Serum was assayed in duplicate in 25 µL allotments using the procedures described by the technical manual from the Diagnostic Systems Laboratories, Inc. (Webster, TX, USA), and values were reported in ng/mL.

Ear tag numbers were transferred to the carcass by writing the number on the left shoulder of each carcass with an edible blue carcass crayon. Carcass order, ear tag number, and tattoo number were documented to match transport, deck, and lairage treatments with blood data. Once carcasses were sorted to a separate rail in the cooler, carcass ear tag ID was transposed onto the left side of the thoracic vertebrae with an edible carcass crayon. After a 20-h chill at 1 °C, carcasses proceeded to fabrication where loins were collected for fresh pork quality data collection.

### 2.2. Pork Quality Measurements

Boneless loins were cut at the 10^th^/11^th^ rib interface, tagged with the original ear tag ID, and moved to a remote table for ultimate pork quality assessment. Ultimate LM pH was measured with a spear-probe attached to a MPI pH-Meter (Meat Probes, Inc., Topeka, KS, USA) and instrumental color scores (L*, a*, and b* values) were measured on the cut surface of the LM after allow a 10-min bloom period [10]. Instrumental color measurements were obtained using a Konica-Minolta portable Chroma Meter (CR 410, Minolta, Osaka, Japan) with D65 illuminant, 10° observer, and the colorimeter was calibrated against a white tile. Chroma ($\sqrt{{\left(a*\right)}^{2}+{\left(b*\right)}^{2}}$ ) and hue angle ($\tan^{-1}\left(b*/a*\right)*180/\pi$ ) were calculated. Chroma (C*), or color saturation index, is a measurement of how vivid or concentrated a color appears (greater C* value indicates a more vivid color), whereas hue angle is a measure of the change in color from the true red axis (greater hue angle indicates a greater change from true red [11]).

A 2.54-cm thick LM chop was fabricated from the 10^th^/11^th^ rib interface, and from the center of these chops, a 2.54-cm diameter core sample was removed for determination of drip loss as described by Rasmussen and Stouffer [12]. Briefly, core samples were weighed (nearest 0.01 g) before being placed into drip loss tubes (C. Christensen Laboratory, Hillerød, Denmark). Drip loss tubes were then placed in a drip loss rack and the entire rack was moved to a 4 °C cooler for 24 h. After 24 h, samples were reweighed. The percentage of moisture loss (24-h drip loss) was calculated by dividing the difference between weights by the initial sample weight and multiplying by 100.

### 2.3. Statistical Analysis

Data were analyzed using the mixed models procedure of SAS (SAS Institute, Cary, NC, USA; version 9.2) with individual hog considered the experimental unit. The model included the fixed effects of season, transport length, deck location, and lairage duration (all 2-, 3-, and 4-way interactions were tested and removed from model if P > 0.1) and the random effect of slaughter day nested within season. Least square means were calculated for all main and interactive effects, and mean separation was accomplished using the PDIFF option with the Tukey-Kramer adjustment, when the F-test was significant (P < 0.05).

## 3. Results and Discussion

### 3.1. Serum Cortisol Concentration

**Figure 4:**
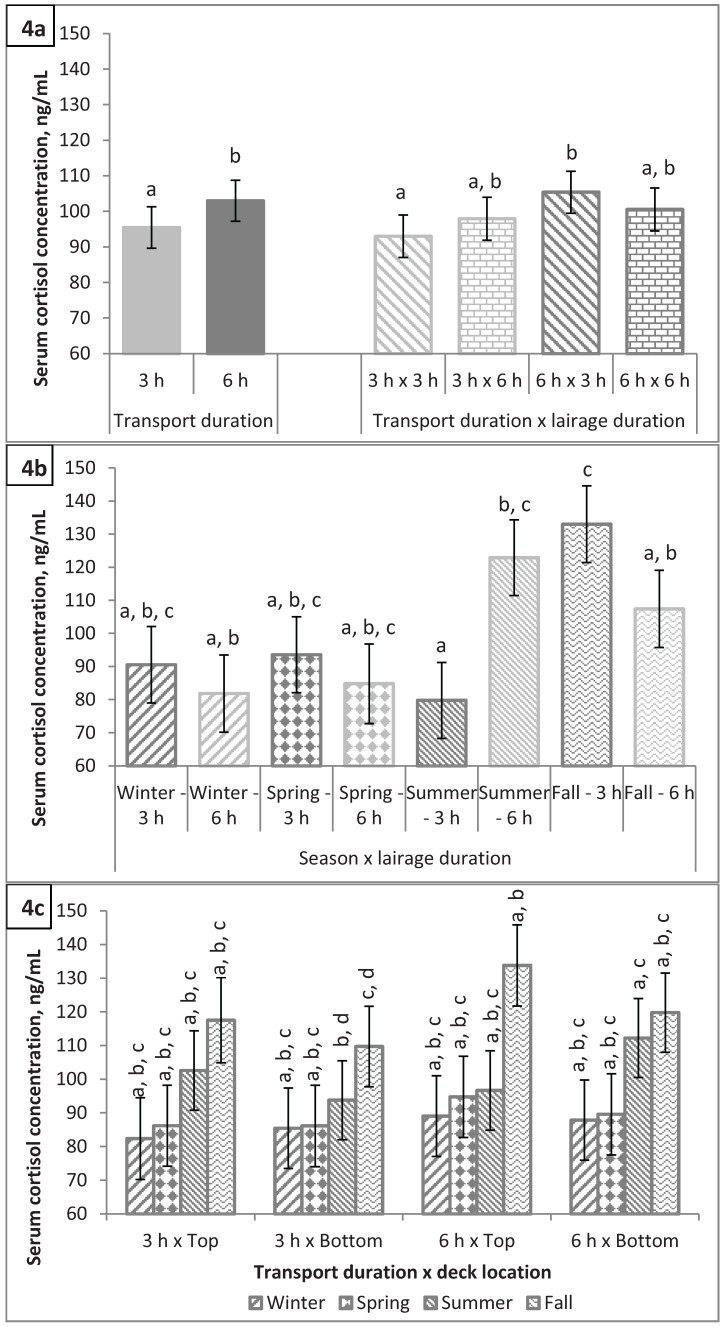
Serum cortisol concentrations (**a**) by transport duration (P = 0.0005) and by transport duration × lairage duration (P = 0.02); (**b**) by season × lairage duration (P < 0.0001); and (**c**) by season × transport duration × deck location (P = 0.02). Values with differing letters differ by P < 0.05.

Cortisol remains active in the body longer than either epinephrine or norepinephrine [13] and is generally regarded as an indicator of the psychological state of an animal, as well as an index of its physiological reaction to environmental conditions and welfare situation [14]. Therefore, measurement of circulating cortisol concentration is common in research which evaluates pre-slaughter stress. Main effects of season, deck location, and lairage duration were not significant for serum cortisol concentration (P > 0.28). Transport duration had a significant (P < 0.001) effect on serum cortisol concentration with hogs being transported for 6 h having a greater serum cortisol concentration than hogs transported for only 3 h (103.0 *vs.* 95.5 ng/mL; Figure 4a). This was driven by the greater serum cortisol concentration in pigs transported for 3 h compared to pigs transported 6 h when lairaged for 3 h (93.0 *vs.* 105.4 ng/mL; P < 0.001) while pigs lairaged for 6 h had similar serum cortisol concentrations regardless of transport duration (97.9 *vs.* 100.5 ng/mL; P = 0.84). Hogs subjected to 3 h of lairage had greater serum cortisol concentrations than hogs subjected to 6 h of lairage in the winter, spring, and fall; however, this was only significant in the fall (P < 0.0001; Figure 4b). In the summer, the opposite was true with hogs subjected to 6 h of lairage having greater serum cortisol concentrations than hogs subjected to 3 h of lairage (122.9 *vs.* 79.7 ng/mL; P < 0.0001). With the exception of hogs transported on the top deck in the summer, hogs transported for 6 h had greater serum cortisol concentrations than hogs transported for 3 h, although this was only significant (P = 0.02) for hogs transported on the bottom deck during the summer (Figure 4c). Although not significant (P > 0.1), hogs transported for 3 h in the summer on the top deck had greater serum cortisol concentrations than hogs transported for 6 h (102.6 *vs.* 96.7 ng/mL).

Martoccia *et al.* [15] concluded that transport distance alone did not determine levels of severe stress in hogs and found that transport stress may be offset by the amount of time hogs spent in lairage before stunning. It is generally accepted that time spent in lairage allows stressed animals to recover from loading, transport, and unloading stress. Grandin [16] recommended that hogs should be rested 2 to 4 h before entering the stunning procedure. The findings of the present study are consistent with Carr *et al.* [17] who demonstrated that, during the heat of the summer months, hogs given a shorter lairage had lower circulating cortisol concentration than hogs held in longer lairage. It is also interesting to note that 3 h lairage after a 6 h haul in our study appeared to induce a much greater stress response as measured by serum cortisol concentration in hogs than those held in 3 h lairage after a 3 h haul (105.4 *vs.* 93.0; P < 0.0001), suggesting that market hogs may require a longer lairage after longer transport duration.

Over 60% of all transport losses occur in less than 30% of the loads of market hogs [18]. This sporadic occurrence is difficult to explain but could be the result of differences in environmental conditions, loading distances at the farm, loading crews, and waiting times at the plant. Ritter *et al.* [18] also reported that internal trailer temperature was not correlated to transport losses and concluded that the variability in death losses among hogs was poorly understood. Cortisol results from the present study indicated an increase in the stress response in the fall that was exacerbated by transport duration and deck location; hogs transported for 6 h on the top deck during the fall had the greatest serum cortisol concentrations (Figure 4c).

### 3.2. Longissimus dorsi 24-h pH

**Figure 5:**
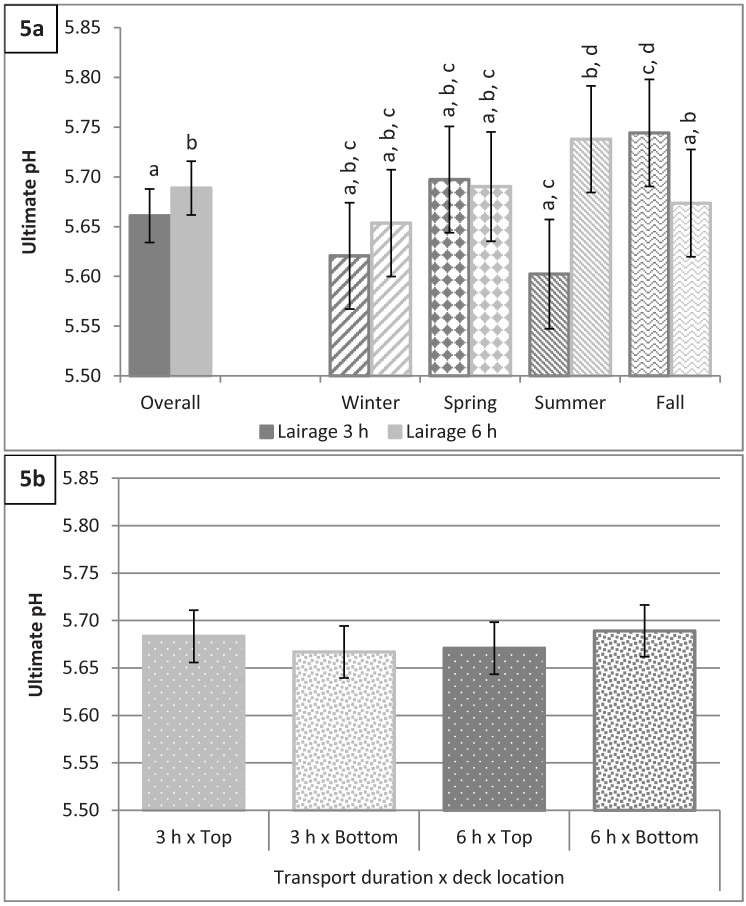
Ultimate pH values (**a**) by lairage duration (P = 0.005) and by season × lairage duration (P < 0.0001) and (**b**) by transport duration × deck location (P = 0.02). Values with different letters differ by P < 0.05.

In the present study, season, deck location, and transport duration did not have a main effect on pH (P > 0.5) while lairage duration had a significant effect (P < 0.01; Figure 5a) on pH with hogs subjected to 3 h of lairage having a lower pH than hogs lairaged for 6 h (5.66 *vs.* 5.69). Significant interactions for pH were season by lairage duration (P < 0.0001; Figure 5a) and deck location by transport duration (P = 0.02; Figure 5b). The pH values for winter and spring were similar (P > 0.25) for hogs lairaged for 3 h *vs.* 6 h. However, pH was increased in hogs lairaged for 6 h in the summer (5.74 *vs.* 5.60; P < 0.0001) and decreased in the fall (5.67 *vs.* 5.74; P < 0.0001) when compared to hogs lairaged for only 3 h. Hogs that were transported for 6 h on the bottom deck tended to have greater pH than hogs transported for 3 h in the bottom deck (5.69 *vs.* 5.67; P = 0.11) while there was no difference in pH between hogs transported for 6 h *vs.* 3 h in the top deck (5.67 *vs.* 5.68; P = 0.63).

Ultimate pH has been shown to be correlated with meat color, tenderness, water holding capacity, and sensory qualities [19,20,21]. Bidner *et al.* [19] showed that greater ultimate pH resulted in improved pork quality as long as ultimate pH did not exceed 6.1. Bidner *et al.* [19] also reported that ultimate pH above 6.2 resulted in DFD meat and below 5.4 in PSE meat. Huff-Lonergan *et al.* [20] showed that ultimate pH was positively correlated with color, firmness, and juiciness score and negatively correlated with percent drip loss and percent cook loss. The results of Huff-Lonergan *et al.* [20] suggest that at higher ultimate pH values, there would be a decreased incidence of PSE. Based on our results, the incidence of PSE meat would be reduced in the summer after a 6 h lairage and in the fall after a 3 h lairage, as these two values were the greatest. 

**Figure 6:**
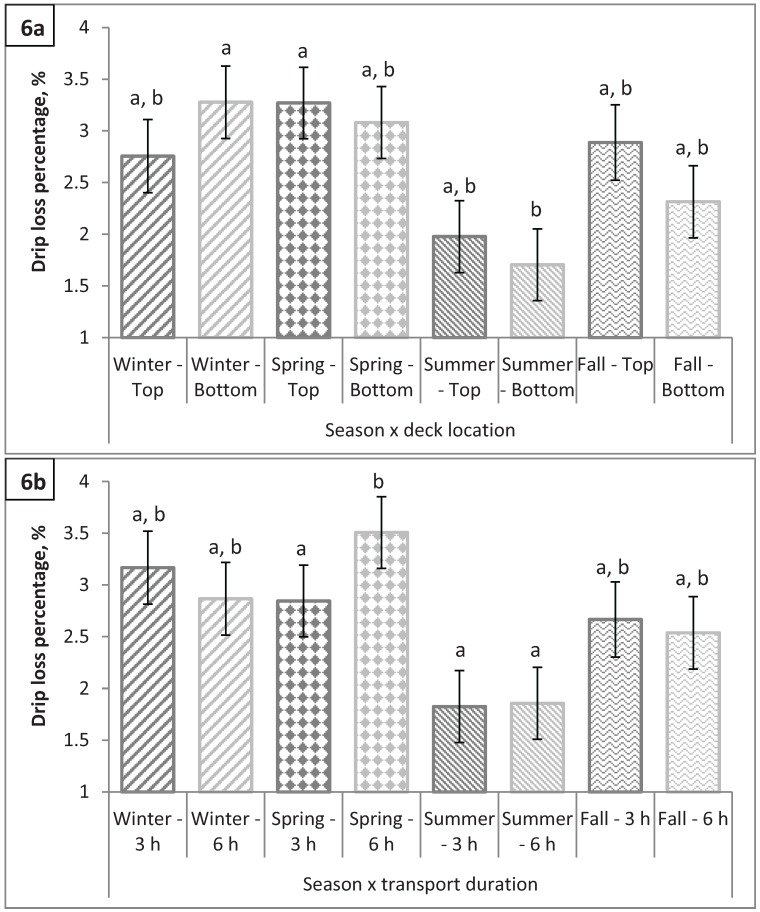
Drip loss percentage (**a**) by season × deck location (P = 0.0002) and (**b**) by season × transport duration (P = 0.0003). Values with different letters differ by P < 0.05.

### 3.3. Longissimus dorsi 24-h Drip Loss Percentage

There were no significant main effects (P > 0.15) for 24-h drip loss percentage; however, two 2-way interactions, season by deck location (P < 0.001; Figure 6a) and season by transport duration (P < 0.001; Figure 6b), were observed for drip loss percentage. Drip loss percentages were similar in spring and summer between hogs transported in the bottom *vs.* top decks (P > 0.6). Hogs transported in the top deck during the fall tended to have greater drip loss percentages than those transported in the bottom deck (2.89 *vs.* 2.31%; P = 0.05) while the opposite held true in the winter with hogs transported in the bottom deck having the greater drip loss percentages when compared to hogs transported in the top deck (3.28 *vs.* 2.76%; P = 0.07). The LM from hogs transported 3 h had lower drip loss percentages than the LM from hogs transported 6 h in the spring (2.84 *vs.* 3.51%; P < 0.001); however, drip loss percentages were not different in the winter, summer, and fall between pigs transported for 3 h *vs.* 6 h (P > 0.7). These interactive effects with season are very difficult to explain. Previous research has shown an increase in DFD pork during the winter months, most likely due to chronic stress (shivering to maintain body temperature) which prevents sufficient lactic acid from reaching the muscles [22]. However, our study showed no difference in drip loss in winter compared to other seasons (P > 0.20).

### 3.4. Longissimus dorsi Color: L*, a*, b*, C*, and Hue Angle

**Figure 7:**
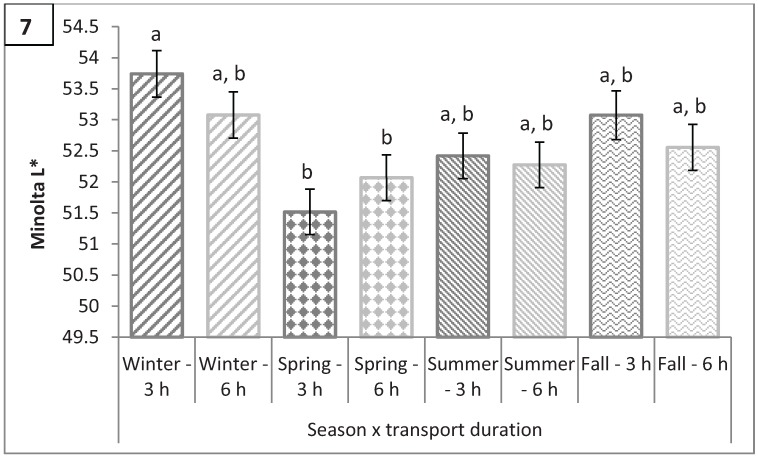
Minolta L* by season × transport duration (P = 0.0005). Values with different letters differ by P < 0.05.

**Figure 8:**
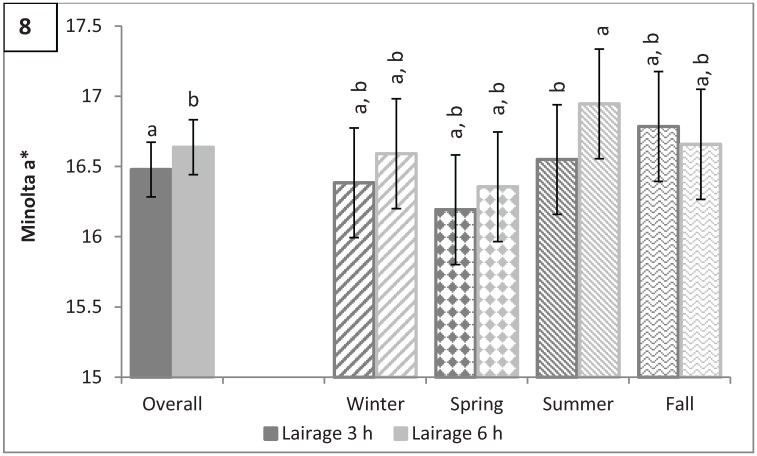
Minolta a* by lairage (P < 0.0001) and by season × lairage duration (P < 0.0001). Values with different letters differ by P < 0.05.

**Figure 9:**
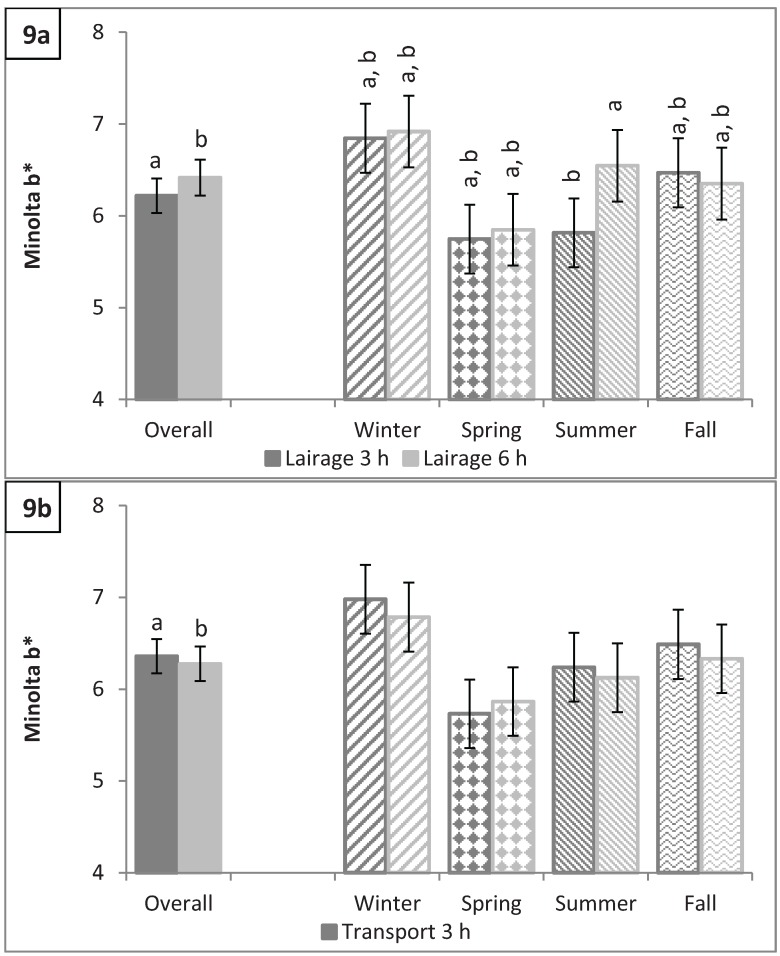
Minolta b* (**a**) by lairage duration (P < 0.0001) and by season × lairage duration (P < 0.0001) and (**b**) by transport duration (P = 0.03) and by season × transport duration (P = 0.009). Values with different letters differ by P < 0.05.

**Figure 10:**
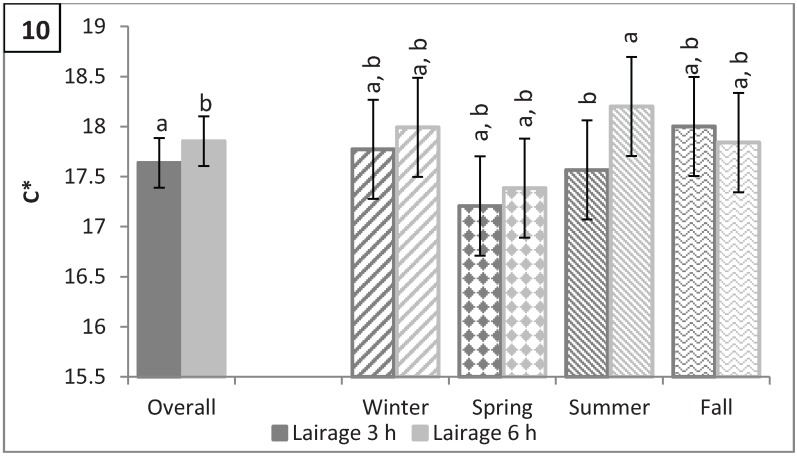
Saturation (C*) by lairage duration (P < 0.0001) and by season × lairage duration (P < 0.0001). Values with different letters differ by P < 0.05.

**Figure 11:**
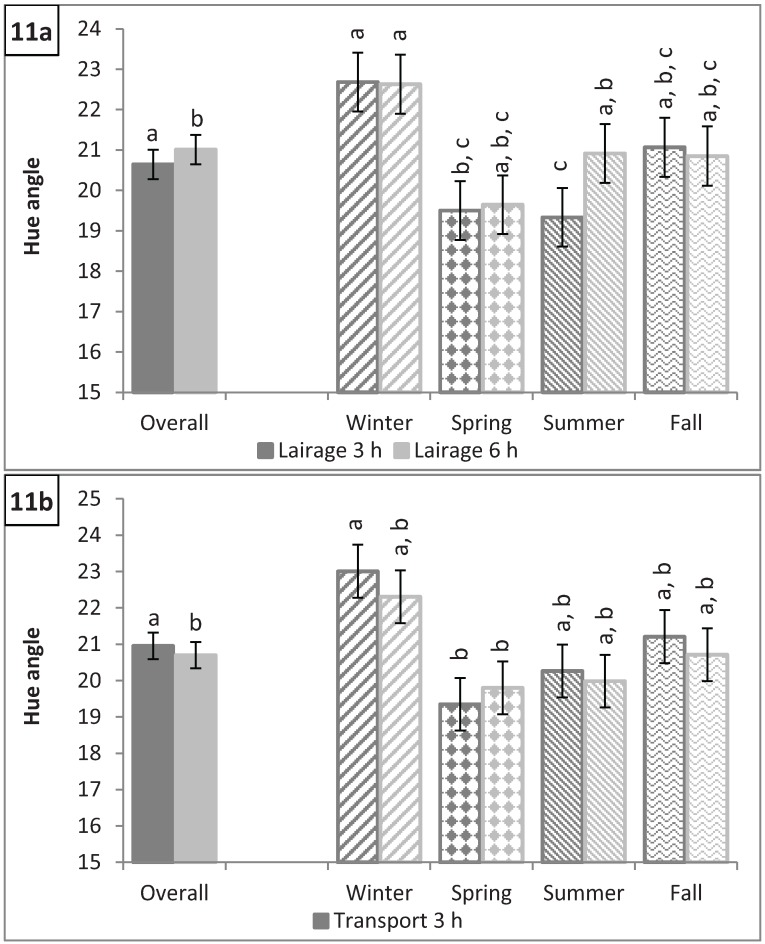
Hue angle (**a**) by lairage duration (P = 0.002) and by season × lairage duration (P < 0.0001) and (**b**) by transport duration (P = 0.03) and by season × transport duration (P = 0.002). Values with different letters differ by P < 0.05.

There was a season by transport duration interaction (P = 0.0005) for L* (Figure 7). The LM of hogs transported for 3 h during the winter tended to be lighter than hogs transported for 6 h (53.74 *vs.* 53.08; P = 0.08). Brewer *et al.* [10] reported that consumers evaluating lightness (L*) values of fresh pork were unable to discern differences in L* values less than 3 units, and, considering statistical differences in L* observed in this study were less than 3 units, results of this study would suggest that there is no observable difference in L* when comparing different transport durations for each season. Lairage duration affected Minolta a*, with hogs lairaged for 3 h having less red pork than hogs lairaged for 6 h (16.48 *vs.* 16.64; P < 0.0001; Figure 8a). The difference in a* between hogs lairaged for 3 h and 6 h was driven by hogs marketed in the summer (16.55 *vs.* 16.95; P < 0.0001) because there was no significant difference in a* in the winter, spring, and fall (P > 0.25). Minolta b* followed the same pattern as a* with hogs lairaged for 6 h in the summer having a greater b* than hogs lairaged for 3 h in the summer (6.55 *vs.* 5.82; P < 0.0001) while there was no significant difference in b* between lairage times in the other seasons (P > 0.85; Figure 9a). Season and transport duration had a significant interaction effect on b* (P = 0.009) although all season by transport duration combinations were not significantly different from each other (P > 0.2; Figure 9b). Lairage duration had the same effect on C* as on a* and b* with hogs being lairaged for 3 h in the summer having a lower C* than hogs lairaged for 6 h in the summer (17.57 *vs.* 18.20; P < 0.0001) while lairage duration did not have a significant effect on C* in the winter, spring, or fall (P > 0.15; Figure 10). Hue angle was also lower for hogs lairaged for 3 h in the summer than those lairaged for 6 h in the summer (19.33 *vs.* 20.91; P < 0.0001); however, unlike a*, b*, and C*, hogs marketed in the winter, regardless of lairage duration, had a greater hue angle than hogs lairaged for 3 h in the spring (P = 0.04) and a tendency for a greater hue angle than hogs lairaged for 6 h in the spring (P = 0.07; Figure 11a). Hogs marketed in the spring, regardless of transport duration, had a lower hue angle than hogs transported for 3 h (P < 0.05) or for 6 h (P < 0.1) in the winter (Figure 11b). While differences in color traits were significant, due to the small differences, it is unlikely that the untrained consumer would notice a difference in color of pork as a result of different transport and lairage durations.

## 4. Conclusions

Pork producers have often been told that stressing hogs can not only have negative consequences on the welfare of hogs but also on the quality of pork that they generate. Removing hogs from their familiar environment, loading them on a trailer, trucking them to a packing plant, unloading them, and leaving them in an unfamiliar pen induces stress on the animal. Cortisol concentration was greatest during the fall season when hogs were in the top deck and transported for a longer period. Consistently, hogs that were transported to slaughter in the summer and winter had higher intramuscular pH after a long lairage, while hogs that had a shorter period of lairage in the spring and fall had higher intramuscular pH. Loins from hogs transported a shorter duration in the spring and summer had significantly lower drip loss than the long transport, while the opposite effect was observed in the fall and winter. Furthermore, higher drip loss was observed in loins from hogs transported on the top deck during spring, summer, and fall; but the opposite during the winter. More research is necessary to determine the factors that deteriorate market hog welfare during some seasons and not others.
